# Overexpression of mGDH in *Gluconobacter oxydans* to improve d-xylonic acid production from corn stover hydrolysate

**DOI:** 10.1186/s12934-022-01763-y

**Published:** 2022-03-09

**Authors:** Xinlei Mao, Baoqi Zhang, Chenxiu Zhao, Jinping Lin, Dongzhi Wei

**Affiliations:** State Key Laboratory of Bioreactor Engineering, New World Institute of Biotechnology, East China University of Science and Technology, Shanghai, 200237 People’s Republic of China

**Keywords:** d-xylonic acid, Lignocellulosic hydrolysate, *Gluconobacter oxydans*, Membrane-bound glucose dehydrogenase

## Abstract

**Background:**

d-Xylonic acid is a versatile platform chemical with broad potential applications as a water reducer and disperser for cement and as a precursor for 1,4-butanediol and 1,2,4-tributantriol. Microbial production of d-xylonic acid with bacteria such as *Gluconobacter oxydans* from inexpensive lignocellulosic feedstock is generally regarded as one of the most promising and cost-effective methods for industrial production. However, high substrate concentrations and hydrolysate inhibitors reduce xylonic acid productivity.

**Results:**

The d-xylonic acid productivity of *G. oxydans* DSM2003 was improved by overexpressing the mGDH gene, which encodes membrane-bound glucose dehydrogenase. Using the mutated plasmids based on pBBR1MCS-5 in our previous work, the recombinant strain *G. oxydans*/pBBR-R3510-mGDH was obtained with a significant improvement in d-xylonic acid production and a strengthened tolerance to hydrolysate inhibitors. The fed-batch biotransformation of d-xylose by this recombinant strain reached a high titer (588.7 g/L), yield (99.4%), and volumetric productivity (8.66 g/L/h). Moreover, up to 246.4 g/L d-xylonic acid was produced directly from corn stover hydrolysate without detoxification at a yield of 98.9% and volumetric productivity of 11.2 g/L/h. In addition, *G. oxydans*/pBBR-R3510-mGDH exhibited a strong tolerance to typical inhibitors, i.e., formic acid, furfural, and 5-hydroxymethylfurfural.

**Conclusion:**

Through overexpressing mgdh in *G. oxydans*, we obtained the recombinant strain *G. oxydans*/pBBR-R3510-mGDH, and it was capable of efficiently producing xylonic acid from corn stover hydrolysate under high inhibitor concentrations. The high d-xylonic acid productivity of *G. oxydans*/pBBR-R3510-mGDH made it an attractive choice for biotechnological production.

**Supplementary Information:**

The online version contains supplementary material available at 10.1186/s12934-022-01763-y.

## Background

d-Xylonic acid (XA) is a versatile platform chemical with broad potential applications, including being a water reducer and disperser for cement [1, 2] and a precursor for copolyamides [3], 1,4-butanediol [4], 1,2,4-tributantriol [5], and 3,4-dihydroxybutyrate [6]. This biobased chemical has been ranked by the U.S. Department of Energy as one of the top 30 high-value chemicals derived from biomass [7].

XA can be produced by enzymatic [8], electrochemical [9] or chemical oxidation [10]. In contrast, microbial conversion of d-xylose to XA has attracted widespread attention and is generally regarded as the most promising method because of its high efficiency. Native XA producers include *Pseudomonas fragi* [11], *Klebsiella pneumoniae* [12], *Enterobacter cloacae* [13], *Paraburkholderia sacchari* [14] and *Gluconobacter oxydans* [15], while other species, such as *Escherichia coli* [16], *Corynebacterium glutamicum* [17], *Pichia kudriavzevii* [18] and *Saccharomyces cerevisiae* [19], have been genetically modified to produce XA. The yield and productivity of XA varied significantly among different strains and their transformation conditions (Additional file 1: Table S1). The highest performance of XA production was observed with *G. oxydans* NL71, which produced 586.3 g/L XA, affording a productivity of 4.69 g/L/h in fed-batch biotransformation with a compressed supply of oxygen [20].

d-Xylose is a highly abundant monosaccharide in nature. It could be generated from the hydrolyzation of lignocellulose materials, which are the most abundant renewable sources [21]. From an economic standpoint, oxidative production of XA from lignocellulose/hemicellulose hydrolysates instead of pure d-xylose is cost-competitive and promising. *G. oxydans* is capable of sugar acid production directly from inexpensive lignocellulosic feedstock. Zhang et al. reported that *G. oxydans* DSM2003 simultaneously produced 132.46 g/L gluconic acid (GA) and 38.86 g/L XA from corn stover hydrolysate (CSH) with biodetoxification [2]. A high-oxygen tension reactor was applied to enhance XA production from the corn stover prehydrolysate without a detoxification process by *G. oxydans* NL71, generating 143.9 g/L XA and affording a volumetric productivity of 1.0 g/L/h [20].

Although *G. oxydans* exhibited more resistance to toxins in biomass hydrolysate than that of other XA-producing strains [2, 15], the presence of relatively high concentrations of hydrolysate inhibitors, i.e., 5-hydroxymethyl furfural (HMF), furfural, 4-hydroxybenzaldehyde, acetic acid, levulinic acid, and vanillin, had adverse effects on XA productivity by *G. oxydans* [20–22]. These degraded chemicals generated from lignocellulose pretreatment act as inhibitors that might inhibit the growth of microorganisms, protein synthesis, and enzyme activity in central metabolism and the target product synthesis pathway [23–26]. Moreover, these inhibitors are likely to become more toxic to microorganisms because they induce a cumulative or synergistic effect despite their very low hydrolysate contents. The critical inhibitory impact of *p*-hydroxybenzaldehyde, formic acid, levulinic acid, and furfural on XA production by *G. oxydans* was reported [20, 27, 28]. Genetic engineering and the adaptive evolution of microorganisms are usually employed to effectively improve microbe resistance and XA production from hydrolysates [27, 29]. For example, overexpressing the thioredoxin gene could enhance the tolerance of* G. oxydans* toward *p*-hydroxybenzaldehyde and formic acid in XA production [27].

In this study, overexpressing membrane-bound glucose dehydrogenase (mGDH), which is able to oxidize d-xylose to XA [30], in *G. oxydans* DSM2003 significantly improved XA production. We found that the typical inhibitors in CSH without detoxification did not impact the d-xylose oxidation efficiency of this recombinant strain. Thus, the effects of five typical lignocellulose-derived inhibitors, namely, formic acid, acetic acid, HMF, furfural, and vanillin, on XA production were investigated. This study provided a potential strain for XA bioproduction from inexpensive d-xylose feedstock, and the fed-batch biotransformation of this strain reached a high XA production.

## Results and discussion

### Overexpression of mgdh in *G. oxydans* DSM2003

The mGDH in *G. oxydans* was capable of catalyzing d-glucose and d-xyloses to the corresponding sugar acids GA and XA, respectively [31]. Overexpression of the membrane-bound PQQ-dependent glucose dehydrogenase gene (*mgdh*) was demonstrated to significantly improve mGDH activity in *G. oxydans* [30]. The precondition for overexpressing an enzyme in a highly efficient manner is an optimized expression plasmid. In our previous study, six new expression plasmids derived from pBBR1MCS-5 (named pBBR-35, pBBR-10, pBBR-R35, pBBR-R10, pBBR-3510, pBBR-R3510) were constructed with higher copy numbers via rational mutagenesis [32]. Herein, to obtain the optimum recombinant strain for XA production, these six mutated plasmids and the origin plasmid pBBR1MCS5 were used to overexpress the mGDH gene in *G. oxydans* DSM2003.

**Fig. 1:**
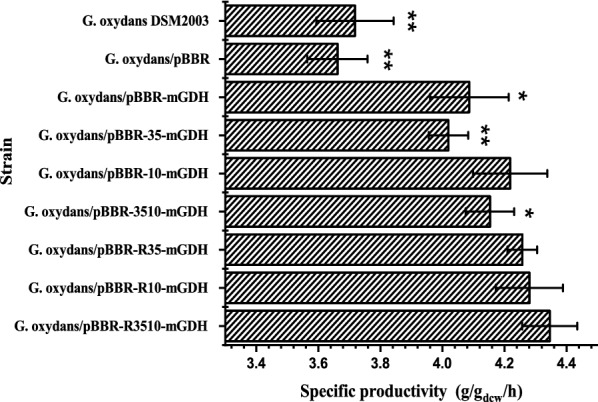
Comparison of specific productivities by different *G. oxydans* strains. The asterisks indicate a significant difference in the specific productivity compared with *G. oxydans*/pBBR-R3510-mGDH (*P < 0.05; and **P < 0.01)

The seven recombinant strains overexpressing *mgdh*, the control strain *G. oxydans*/pBBR, and the parent *G. oxydans* DSM2003 were used to catalyze d-xylose in shaking flasks. The specific productivities of these strains were compared (Fig. 1). All of the *mgdh*-overexpressing strains showed higher specific productivities of XA than those of the control strain *G. oxydans*/pBBR and the parental strain *G. oxydans* DSM2003 (*P* < 0.05). Among these recombinant strains, *G. oxydans*/pBBR-R3510-mGDH, *G. oxydans*/pBBR-R10-mGDH, and *G. oxydans*/pBBR-R35-mGDH exhibited the highest specific productivities (4.35 ± 0.09, 4.28 ± 0.11, and 4.26 ± 0.05 g/g_dcw_/h), which were approximately 15% higher than that of *G. oxydans* DSM2003. Comparatively, *G. oxydans*/pBBR-R3510-mGDH exhibited the highest specific productivity and was chosen for further study. The transcription level of *mgdh* in *G. oxydans*/pBBR-R3510-mGDH was significantly upregulated, as analyzed via quantitative real-time PCR (real-time qPCR), and was 58.55 ± 5.72-fold higher than that in *G. oxydans* DSM2003.

**Fig. 2:**
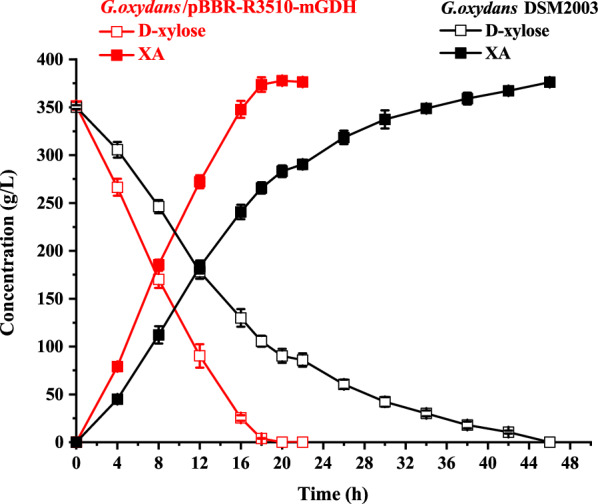
Batch conversion of d-xylose to XA by *G. oxydans*/pBBR-R3510-mGDH and the control *G. oxydans* DSM2003

To further assess the enhancement of XA production of *G. oxydans*/pBBR-R3510-mGDH, d-xylose bioconversion was performed in a 7 L fermenter that allowed for the aeration rate and pH value to be accurately controlled. The reaction system contained 350 g/L d-xylose and 3.82 g_dcw_/L resting cells of *G. oxydans*/pBBR-R3510-mGDH or the parent *G. oxydans* DSM2003. For batch bioconversion by *G. oxydans*/pBBR-R3510-mGDH (Fig. 2), XA accumulation increased rapidly to the maximum titer of 377.8 ± 4.2 g/L in 20 h, affording a volumetric productivity of 18.89 g/L/h and a specific productivity of 4.95 g/g_dcw_/h. In contrast, the parent *G. oxydans* DSM2003 needed more time (46 h) to completely convert 350 g/L d-xylose, affording a volumetric productivity of 8.18 g/L/h and a specific productivity of 2.14 g/g_dcw_/h. Thus, the overexpression of *mgdh* in *G. oxydans* with the modification plasmid pBBR-R3510 significantly enhanced d-xylose oxidation, leading to an improved specific productivity of XA, which was increased by approximately 131% compared to that of the parent *G. oxydans* DSM2003.

**Fig. 3:**
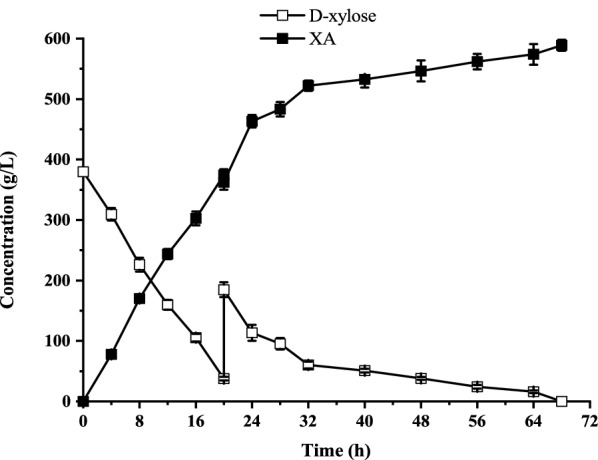
Fed-batch conversion of 535 g/L d-xylose by *G. oxydans*/pBBR-R3510-mGDH

To explore the potential of *G. oxydans*/pBBR-R3510-mGDH for XA production and achieve a higher XA titer, the initial substrate concentration was increased to 535 g/L. In batch bioconversion, 3.82 g_dcw_/L resting cells of *G. oxydans*/pBBR-R3510-mGDH could not completely exhaust the supplied d-xylose, although the conversion time was extended to 65 h (Additional file 1: Fig. S2), suggesting that high concentrations of d-xylose may cause adverse effects. Thus, the fed-batch operation was conducted with an initial 380 g/L d-xylose and 3.82 g_dcw_/L resting cells of *G. oxydans*/pBBR-R3510-mGDH (Fig. 3). Solid d-xylose (310 g) was added when the d-xylose concentration in the reaction system was below 40 g/L. A total of 535 g/L d-xylose was completely exhausted, and the XA titer reached a maximum of 588.7 g/L at 68 h, resulting in a specific productivity of 2.27 g/g_dcw_/h, yield of 99.4% and record high volumetric productivity of 8.66 g/L/h (Additional file 1: Table S1).

### d-Xylonic acid production from corn stover hydrolysate

**Fig. 4:**
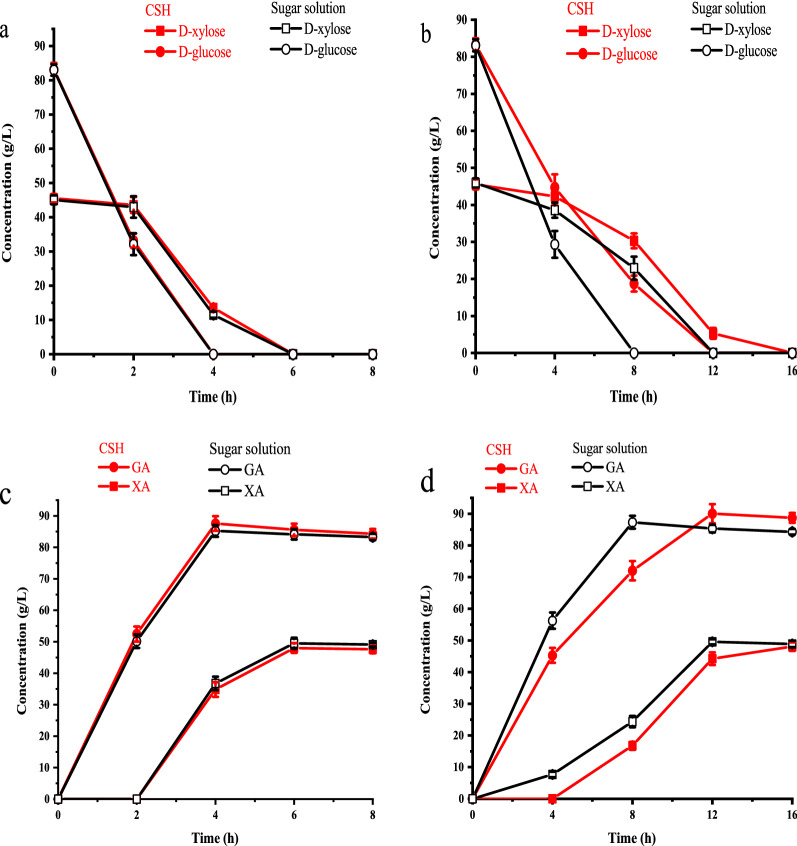
Comparison of biocatalyst performance between CSH and sugar solution. **a** d-Xylose and d-glucose conversion by *G. oxydans*/pBBR-R3510-mGDH. **b** d-xylose and d-glucose conversion by *G. oxydans* DSM2003. **c** XA and GA production by *G. oxydans*/pBBR-R3510-mGDH. **d** XA and GA production by *G. oxydans* DSM2003

To evaluate the potential application of *G. oxydans*/pBBR-R3510-mGDH in XA production from lignocellulose, the conversion performance of this strain was evaluated in CSH without biodetoxification. CSH was prepared by a dry dilute acid pretreatment and enzymatic hydrolysis using cellulase [33, 34]. The experimental CSH contained 83 g/L d-glucose, 45 g/L d-xylose, and inhibitors (i.e., 2.9 g/L acetic acid, 0.48 g/L formic acid, 0.23 g/L furfural, 0.21 g/L HMF, and 0.1 g/L vanillin). The bioconversion of pure sugar solution containing the same concentrations of d-xylose and d-glucose was carried out as the control. While the mixed sugar conversion was performed by the recombinant and parental strains, d-glucose and d-xylose were simultaneously transformed to GA and XA, and the conversion rate of d-glucose was higher than the d-xylose conversion rate (Fig. 4). As anticipated, the conversion rates of d-glucose to GA and d-xylose to XA of *G. oxydans*/pBBR-R3510-mGDH were significantly improved compared with those of the parent *G. oxydans* DSM2003. Hence, the total conversion time was decreased from 16 to 6 h. It was noted that the sugar conversion in CSH by *G. oxydans*/pBBR-R3510-mGDH was essentially the same as that in the pure sugar solution (Fig. 4a), while both the d-glucose conversion rate and d-xylose conversion rate of CSH by *G. oxydans* DSM2003 were reduced (Fig. 4b). These results showed that the sugar (d-xylose and d-glucose) oxidation efficiency of the obtained recombinant strain was nearly independent of the inhibitor disturbance in the experimental CSH.

### The influence of inhibitors derived from lignocellulose on D-xylonic acid production

To explore the potential tolerance of *G. oxydans*/pBBR-R3510-mGDH to lignocellulose-derived inhibitors, three types of compounds that are commonly found in lignocellulosic hydrolysates were selected to further assess their influence on XA productivity by resting cells. The three types of compounds investigated included organic acids (formic acid and acetic acid), furan aldehydes (HMF and furfural), and an aromatic compound (vanillin). The impact of inhibitors on the parental strain *G. oxydans* DSM2003 was conducted as the control.

**Fig. 5:**
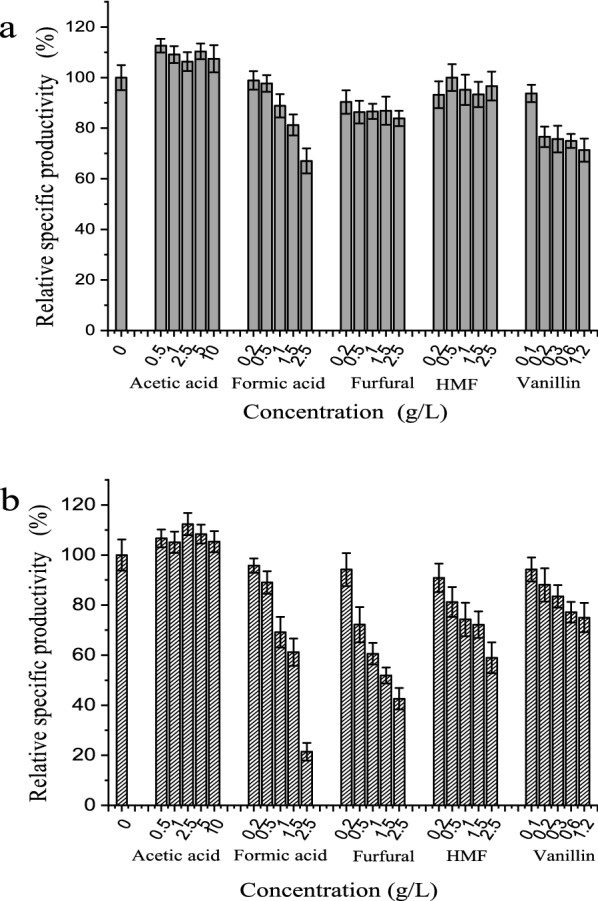
Relative specific productivities in synthetic medium with different inhibitor contents. **a** *G. oxydans*/pBBR-R3510-mGDH. **b** *G. oxydans* DSM2003

As an acetic acid bacterium, *G. oxydans* DSM 2003 was strongly tolerant to acetic acid [35, 36]. As shown in Fig. 5, the XA productivity of *G. oxydans*/pBBR-R3510-mGDH or *G. oxydans* DSM2003 was essentially not influenced by acetic acid in the concentration range studied (below 10 g/L). Acetate was a typical metabolite in the culture of *G. oxydans*. Vanillin below 0.1 g/L did not severely inhibit XA production by *G. oxydans*/pBBR-R3510-mGDH or *G. oxydans* DSM2003. If the concentration of vanillin was increased to 1.2 g/L, the XA productivities of *G. oxydans*/pBBR-R3510-mGDH and *G. oxydans* DSM2003 decreased by 71.3% and 75.0%, respectively.

Figure 5 indicates that the mGDH-overexpressing strain *G. oxydans*/pBBR-R3510-mGDH exhibited better resistance to formic acid, furfural, and HMF than that of the parental strain *G. oxydans* DSM2003. For *G. oxydans*/pBBR-R3510-mGDH, formic acid within the concentration range of 0.2–0.5 g/L did not exhibit a clear negative impact on XA production. However, XA productivity slightly decreased at higher formic acid concentrations. In contrast, the XA productivity of *G. oxydans* was strongly affected by formic acid within the concentration range studied (0.2–2.5 g/L). Although the XA productivity of *G. oxydans* DSM2003 declined as the concentration of furfural or HMF increased, the XA productivity of *G. oxydans*/pBBR-R3510-mGDH remained almost constant at varying concentrations of furfural or HMF, even under the high concentration of 2.5 g/L, which reached inhibitory concentrations that were lethal to some microorganisms utilized in the lignocellulose biotransformation [37–39].

These results indicated that the overexpression of mGDH responsible for D-xylose oxidation efficiently enhanced the XA productivity of *G. oxydans* DSM2003 and strengthened the resistance to inhibitors from lignocellulose degradation. The high abundance of mGDH and the robust activity of mGDH toward typical lignocellulose-derived inhibitors should be one explanation for the improved XA productivity of *G. oxydans* from lignocellulose (i.e., corn stover) hydrolysate. According to reports, *G. oxydans* exhibits a strong tolerance to toxins in lignocellulose hydrolysate as they rapidly transform into less toxic metabolites [2]. For example, HMF and furfural could be transformed to 5-hydroxymethyl-2-furoic acid and furoic acid by *G. oxydans* [40, 41]. We also found that overexpressing mGDH in *G. oxydans* significantly accelerated the conversion of furfural or HMF (Additional file 1: Fig. S4). In addition, the membrane fraction of *G. oxydans*/pBBR-R3510-mGDH showed higher dehydrogenation activity toward furfural or HMF than that of the parental strain (Additional file 1: Fig. S5). These results demonstrated that membrane-bound dehydrogenases in *G. oxydans* played a role in rendering *G. oxydans* tolerant to furfural and HMF and implied that mGDH could catalyze furfural and HMF to the corresponding furoic acid and 5-hydroxymethyl-2-furoic acid. Acetic acid bacteria, including *G. oxydans*, are tolerant to organic acids (i.e., formic acid and acetic acid) because they can pump out protons to diminish the adverse effects of organic acids [42]. During this process, membrane-bound PQQ-dependent dehydrogenases played a role in providing energy for the process of pumping out protons. Thus, overexpressing PQQ-dependent mGDH might enhance the energy supply to pump out protons and render *G. oxydans* tolerant to formic acid.

### d-Xylonic acid production from corn stover hydrolysate containing more d-xylose and inhibitors by *G. oxydans*/pBBR-R3510-mGDH

**Fig. 6:**
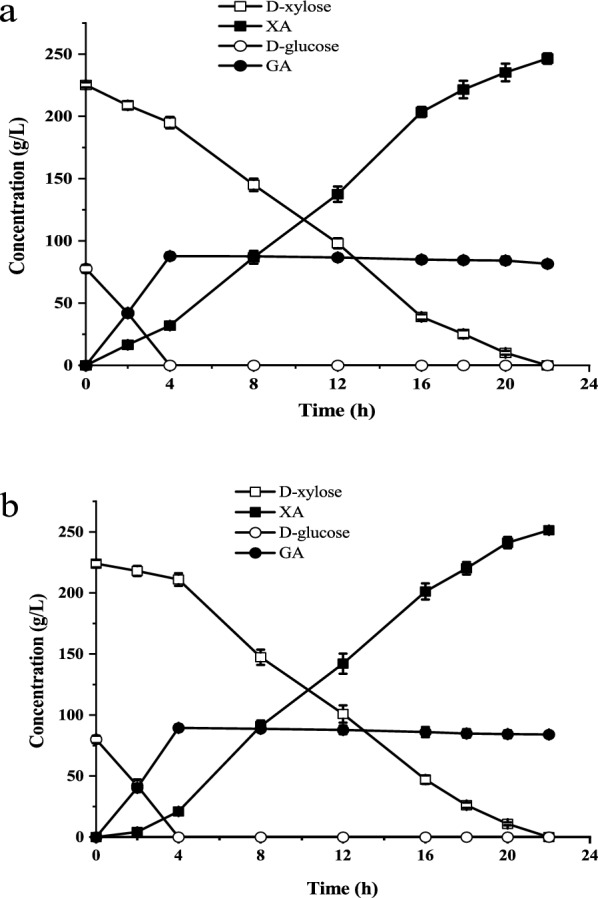
XA and GA production under high concentrations of inhibitors by *G. oxydans*/pBBR-R3510-mGDH in the fermenter. **a** Conversion of CSH containing more d-xylose and inhibitors. **b** Conversion of sugar solution

The production of XA by *G. oxydans*/pBBR-R3510-mGDH from CSH that contained more D-xylose and inhibitors was also tested in the fermenter. This hydrolysate was prepared by adding xylose and inhibitors to CSH and contained 225.3 g/L d-xylose, 77.6 g/L d-glucose, and five high concentrations of inhibitors, i.e., 10 g/L acetic acid, 1.5 g/L formic acid, 2.5 g/L furfural, 2.5 g/L HMF and 0.1 g/L vanillin. Figure 6a shows that both d-xylose and d-glucose in the hydrolysate were efficiently and completely converted by the recombinant strain *G. oxydans*/pBBR-R3510-mGDH. GA production remained considerably high with a GA concentration of 81.60 g/L, affording a GA yield of 96.5%. In addition, 225.3 g/L d-xylose was converted to 246.4 g/L XA in 22 h with a yield of 98.9%. Similar XA and GA production was found in the conversion of the pure sugar solution (Fig. 6b). The total volumetric productivity of XA and GA in the hydrolysate conversion was 14.91 g/L/h. This value was similar to the total volumetric productivity (15.25 g/L/h) in the conversion of the pure sugar solution. All results indicated that *G. oxydans*/pBBR-R3510-mGDH possessed an excellent ability for CSH bioconversion, even if the total amount of the five inhibitors reached 16.6 g/L. The recombinant strain *G. oxydans*/pBBR-R3510-mGDH is a competitive biocatalyst for producing high amounts of sugar acid from lignocellulose feedstock.

## Conclusion

In this study, by overexpressing mGDH in *G. oxydans* DSM2003, a significant improvement in XA production was successfully achieved. XA production with the recombinant strain *G. oxydans*/pBBR-R3510-mGDH was characterized by a maximum product yield and a high volumetric productivity under batch and fed-batch processes that utilized D-xylose and corn stover hydrolysate, respectively. A detailed analysis of the influence of lignocellulose-derived inhibitors revealed that overexpression of mGDH was considered to play a possible role in rendering *G. oxydans* tolerant to formic acid, furfural and HMF.

## Materials and methods

### Organisms and culture conditions

Sorbitol medium consisting of 80 g/L sorbitol, 20 g/L yeast exact, 1 g/L KH_2_PO_4_, 0.3 g/LMgSO_4_, and 0.1 g/L glutamine was used for *G. oxydans* strain cultivation with an initial pH of 6.0 at 30 °C in shaking flasks or in a 7 L fermenter. Luria−Bertani medium was used for recombinant *E. coli* strain cultivation at 37 °C and 200 rpm. To maintain plasmids, the concentration of gentamicin or kanamycin in the medium was 25 μg/L.

### Plasmid and strain construction

The primers used in this work are shown in Additional file 1: Table S2. To overexpress mGDH in *G. oxydans* DSM2003, fragments of the *mgdh* gene encoding mGDH and promoter *tufb* were amplified from the *G. oxydans* DSM2003 genome by using the primer pair gdh-f/gdh-r and tufb-f/tufb-r. The plasmid pBBR1MCS5 and the fragment of promoter *tufb* were digested by endonucleases *Xba*I and *Sac*I and then ligated by T4 ligase to construct the plasmid pBBR-P*tufb*. The fragment of *mgdh* and the plasmid pBBR-P*tufb* were digested by endonucleases *Xba*I and *Eco*RI and then ligated by T4 ligase to construct the recombinant plasmid pBBR-P*tufb*-mGDH that was used for overexpressing mGDH. The other six recombinant plasmids, pBBR-35-P*tufb*-mGDH, pBBR-10-P*tufb*-mGDH, pBBR-R35-P*tufb*-mGDH, pBBR-R10-P*tufb*-mGDH, pBBR-3510-P*tufb*-mGDH, and pBBR-R3510-P*tufb*-mGDH, were constructed in the same way.

Triparental mating was applied to transform the recombinant plasmids to overexpress and the control plasmid pBBR-P*tufb* into *G. oxydans* DSM2003 [43].

### Quantitative real-time PCR

Real-time qPCR was applied to analyze the gene transcription level in *G. oxydans* strains. Reagents and enzymes were purchased from Transgene (China) and used for RNA isolation and cDNA synthesis. GenScript Real-time PCR (TaqMan) Primer Design was utilized to design the primers used in real-time qPCR. The cells were cultivated in sorbitol medium for the late exponential phase and then harvested for RNA isolation. cDNA was synthesized, and the transcription level of *mgdh* was measured following the manufacturer’s instructions. The 16S rRNA of *G. oxydans* DSM2003 was applied as an internal control. All experiments were done in triplicate. Three parallel experiments were performed. The 2^−△△Ct^ method was used to analyze the data.

### Conversion of d-xylose by *G. oxydans* in shake flasks

The level of XA production by *G. oxydans* was evaluated by examining the production of XA in 2 h. *G. oxydans* strains were cultivated to the late exponential phase, collected by centrifugation at 8000 rpm for 8 min and washed with 50 mM citric acid buffer (pH 5.8) once to obtain resting cells. The reaction system contained 40 g/L d-xylose, 50 mM citric acid buffer (pH 5.8), and 1.90 g_dcw_/L resting cells in shake flasks at 30 °C and 200 rpm. All the experiments were performed in triplicate.

### Preparation of the corn stover hydrolysate

CSH without detoxification was provided by Prof. Jie Bao and Dr. Jian Zhang. Corn stover was preprocessed by a dry dilute acid pretreatment and then hydrolyzed by cellulase to obtain the CSH without detoxification [33, 34]. The CSH slurry was centrifuged at 8000 rpm for 15 min to remove the solids. The CSH supernatant, which contained 83 g/L d-glucose, 45 g/L d-xylose, 2.9 g/L acetic acid, 0.48 g/L formic acid, 0.23 g/L furfural, 0.21 g/L HMF, and 0.1 g/L vanillin, was utilized for bioconversion. The CSH containing more d-xylose and inhibitors was prepared by adding d-xylose and inhibitors to the CSH, which contained 225.3 g/L d-xylose, 77.6 g/L d-glucose, 10 g/L acetic acid, 1.5 g/L formic acid, 2.5 g/L furfural, 2.5 g/L HMF and 0.1 g/L vanillin.

### Whole-cell transformation in the fermenter

Whole-cell transformation in the batch was performed in a 7 L fermenter with 4 vvm aeration at 30 °C and 600 rpm. A 4 M NaOH solution was used to control the pH in the reaction system at 5.8. The reaction system consisted of resting cells and a D-xylose solution/hydrolysate/sugar solution. The concentration of resting cells in the reaction was 3.82 g_dcw_/L.

Whole-cell transformation in the fed-batch was also carried out in a 7 L fermenter. The working volume was 2 L. The conditions were consistent with those in the batch transformation. The initial reaction system contained 380 g/L d-xylose and 3.82 g_dcw_/L resting cells. In 22 h, 310 g of solid d-xylose was fed into the reaction system, and the total loading concentration of d-xylose reached 535 g/L. Two parallel experiment groups were carried out.

Samples were taken at 4 h intervals and centrifuged at 12,000 rpm for 10 min to obtain the supernatant used to analyze substrates and products.

### d-Xylonic acid production in synthetic medium with different inhibitor contents

The influence of inhibitors on XA production was evaluated by the relative specific productivity of XA in synthetic medium with different inhibitor contents. The synthetic medium contained 40 g/L d-xylose, 1.90 g_dcw_/L resting cells, 50 mM citric acid buffer (pH 5.8) and different contents of acetic acid, formic acid, furfural, HMF, and vanillin. All experiments involved three parallel groups. The specific productivity from the synthetic medium without inhibitors was determined to be 100%.

### Analysis of substrates, products, and inhibitors

Before analysis, 0.22 μm membrane filters were used to filter samples. Substrates (d-xylose and d-glucose) were measured by HPLC (1260 Infinity, Agilent Technologies, USA) equipped with an Agilent ZORBAX Carbohydrate Analysis column and detected by a refractive index detector (G1362A, Agilent Technologies, USA). The temperature of the oven was maintained at 30 °C. The mobile phase was a 70% (v/v) acetonitrile–water solution. The flow rate was 1 mL/min.

XA and GA were analyzed by HPLC with a Bio–Rad Aminex HPX-87H column at 55 °C, and detected by a UV–VIS detector (G7114A, Agilent Technologies, USA) at 210 nm. The mobile phase was 5 mM H_2_SO_4_. The flow rate was 0.4 mL/min.

Vanillin was measured by HPLC with a YM-Pack ODS-A column (YMC, Japan) and detected at 270 nm by a UV–VIS detector. The oven temperature was 35 °C, and acetonitrile and a 0.1% (v/v) formic acid solution were used for the mobile phase. The acetonitrile content in the mobile phase was adjusted as described in Khoddami’s report [44]. The flow rate was 1.0 mL/min.

A Bio–Rad Aminex HPX-87H column was used to analyze furfural, HMF, acetic acid, and formic acid by HPLC with a refractive index detector (G1362A, Agilent Technologies, USA). The oven temperature was 65 °C. The mobile phase was 5 mM H_2_SO_4_. The flow rate was 0.6 mL/min.

The specific productivity of XA was determined by the titer of XA and the concentration of resting cells in the reaction system and was calculated by using Eq. (1).

1 $$\text{Specific productivity }\left(\text{g/g}{\text{dcw}}\text{/h}\right) = \frac{\left[XA\right]}{\left[resting cells\right]*\left[time\right]},$$

where [*XA*] (g/L) is the concentration of XA; [*resting cells*] (g_dcw_/L) is the concentration of resting cells in the reaction; and [*time*] (h) is the reaction time.

The yield was determined by the ratio between the amount of the substrate converted into the product and the amount of loaded substrate and calculated by using Eq. (2).

2 $${\text{Yield}}\left({\%}\right)=\frac{\left[Product\right]-{\left[Product\right]}_{0}}{{\mathrm{F}}*{\left[Substrate\right]}_{0}}*100{\%},$$

where [*Product*] (g/L) is the concentration of product at the end; [*Product*]_0_ (g/L) is the initial concentration of product; [*Substrate*]_0_ (g/L) is the initial concentration of substrate; F is the factor of substrate converted to an equal product depending on the stoichiometric balance. The value of F used in the yield of XA was 1.11. The value of F used in the yield of GA was 1.09.

The volumetric productivity was determined by the product titer at the end of the reaction and was calculated by using Eq. (3).

3 $${\text{Volumetric productivity}}\left({{\rm g}/{\rm L}/{\rm h}}\right) = \left[Product\right]/\left[time\right],$$

where [*Product*] (g/L) is the concentration of product at the end of the reaction; [*time*] (h) is the reaction time at the end.

## Supplementary Information

**Additional file 1: Table S1.** Comparison of d-xylonic acid production by different organisms. **Table S2**. Strains, plasmids and primers used in this study. **Figure S1.** Comparison of d-xylonic acid production by different *G. oxydans* strains. The conversions were carried out in shake flasks at 30 °C and 200 rpm. The reaction system contained 40 g/L d-xylose, 50 mM citric acid buffer (pH 5.8) and 1.90 g_dcw_/L resting cells. **Figure S2.** The activities of mGDHs from *G. oxyd*ans DSM2003 and *G. oxydans*/pBBR-R3510-mGDH toward xylose. **Figure S3.** Batch D-xylonic acid production from 535 g/L d-xylose by *G. oxydans*/pBBR-R3510-mGDH. The conversion was carried out in a 7 L fermenter with 4 vvm aeration, 30 °C and 600 rpm conditions. The pH was maintained at 5.8 by using a 4 M NaOH solution. The reaction system contained 535 g/L d-xylose and 3.82 g_dcw_/L resting cells. **Figure S4.** Transformation of furfural and HMF by *G. oxydans*/pBBR-R3510-mGDH and *G. oxydans* DSM2003, respectively. (a) Transformation of furfural; (b) transformation of HMF. The conversions were carried out in shake flasks at 30 °C and 200 rpm. The reaction system contained 0.5 or 2.5 g/L furfural (HMF), 40 g/L d-xylose, 50 mM citric acid buffer (pH 5.8), and 1.90 g_dcw_/L resting cells. **Figure S5.** The activities of the membrane-bound dehydrogenases of membrane fractions from *G. oxydans* DSM2003 and *G. oxydans*/pBBR-R3510-mGDH toward furfural and HMF.

## Data Availability

The datasets used and analyzed during the current study are available from the corresponding author upon reasonable request.

## Abbreviations

XA: Xylonic acid
GA: Gluconic acid
HMF: 5-Hydroxymethyl furfural
mGDH: Membrane-bound glucose dehydrogenase enzyme
*mgdh*: Membrane-bound glucose dehydrogenase gene
CSH: Corn stover hydrolysate
real-time qPCR: Quantitative real-time PCR
dcw: Dry cell weight
