# Supplementary material for: Overexpression of mGDH in Gluconobacter oxydans to improve d-xylonic acid production from corn stover hydrolysate
Source: Microb Cell Fact. 2022 Mar 9;21:35. doi: 10.1186/s12934-022-01763-y (PMC8905809; doi:10.1186/s12934-022-01763-y)
Supplement: Supplementary file 1 — Additional file 1: Table S1. Comparison of d-xylonic acid production by different organisms. Table S2. Strains, plasmids and primers used in this study. Figure S1. Comparison of d-xylonic acid production by different G. oxydans strains. The conversions were carried out in shake flasks at 30 °C and 200 rpm. The reaction system contained 40 g/L d-xylose, 50 mM citric acid buffer (pH 5.8) and 1.90 gdcw/L resting cells. Figure S2. The activities of mGDHs from G. oxydans DSM2003 and G. oxydans/pBBR-R3510-mGDH toward xylose. Figure S3. Batch D-xylonic acid production from 535 g/L d-xylose by G. oxydans/pBBR-R3510-mGDH. The conversion was carried out in a 7 L fermenter with 4 vvm aeration, 30 °C and 600 rpm conditions. The pH was maintained at 5.8 by using a 4 M NaOH solution. The reaction system contained 535 g/L d-xylose and 3.82 gdcw/L resting cells. Figure S4. Transformation of furfural and HMF by G. oxydans/pBBR-R3510-mGDH and G. oxydans DSM2003, respectively. (a) Transformation of furfural; (b) transformation of HMF. The conversions were carried out in shake flasks at 30 °C and 200 rpm. The reaction system contained 0.5 or 2.5 g/L furfural (HMF), 40 g/L d-xylose, 50 mM citric acid buffer (pH 5.8), and 1.90 gdcw/L resting cells. Figure S5. The activities of the membrane-bound dehydrogenases of membrane fractions from G. oxydans DSM2003 and G. oxydans/pBBR-R3510-mGDH toward furfural and HMF. [file 12934_2022_1763_MOESM1_ESM.docx]

**Additional file 1**

**Overexpression of mGDH in Gluconobacter oxydans to improve D-xylonic acid production from corn stover hydrolysate**

**Table S1.** Comparison of D-xylonic acid production by different organisms

| **Strain** | **Feedstock** | **Titer**  **(g/L)** | **Yield**  **(%)** | **Volumetric productivity (g/L/h)** | **Specific productivity**  **(g/g_dcw_/h)** | **Process** | **Aerate** | **Reference** |
| --- | --- | --- | --- | --- | --- | --- | --- | --- |
| *G. oxydans* DSM 2003 | Corn stover hydrolysate | 38.86 | 87.72 | 1.21 | — | Fermentation. | Air | Zhang et al., [1] |
| *G. oxydans* NL71 | D-Xylose | 586.3 | 88.30 | 4.69 | — | Whole-cell catalysis; Fed batch. | Oxygen | Zhou et al., [2] |
| *G. oxydans* NL71 | Corn stover hydrolysate | 186 | 87.45 | 0.97 | — | Whole-cell catalysis. | Oxygen | Zhou et al., [2] |
| *G. oxydans* NL71 | Corncob prehydrolysate | 143.6 | 91.96 | 4.48 | 1.12 | Whole-cell catalysis. | Air | Dai et al., [3] |
| *Paraburkholderia sacchari* | D-Xylose | 373.5 | ~100 | 4.42 | ~0.18 | Fed-batch fermentation. | Air | Bondar et al., [4] |
| *Pseudomonas putida* | D-Xylose | 8.5 | 76.8 | 0.56 | — | Whole-cell catalysis. | Air | Dvořák et al., [5] |
| *Candida glycerinogenes* | D-Xylose | 38 | 68.7 | 0.79 | 0.16 | Fermentation. | Air | Ji et al., [6] |
| *Pseudomonas fragi* | D-Xylose | 162 | 97.6 | 1.4 | 0.7 | Fermentation. | Air | Buchert and Viikari, [7] |
| *Pichia kudriavzevii* | D-Xylose | 171 | 90.36 | 1.4(initial) | 0.11 | Fed-batch fermentation. | Air | Toivari et al., [8] |
| *Corynebacterium glutamicum* | D-Xylose | 56.32 | 84.82 | 0.47 | 0.34 | Fermentation. | Air | Sundar et al., [9] |
| *Corynebacterium glutamicum* | Rice straw hydrolysate | 42.94 | 64.67 | 0.36 | — | Fermentation. | Air | Sundar et al., [9] |
| *E. coli* | D-Xylose | 199.44 | 85.82 | 7.12 | — | Fed-batch fermentation. | Air | Gao et al., [10] |
| *E. coli* | Corn stover hydrolysate | 91.2 | 95.05 | 1.52 | — | Fed-batch fermentation. | Air | Zhang et al., [11] |
| *Saccharomy cescerevisiae* | D-Xylose | 49 | 102.97 | 0.44 | 0.064 | Fermentation. | Air | Toivari et al., [12] |
| *G. oxydans*/pBBR-R3510-mGDH | D-Xylose | 588.7 | 99.43 | 8.66 | 2.27 | Whole-cell catalysis; Fed-batch. | Air | This study |
| *G. oxydans*/pBBR-R3510-mGDH | Corn stover hydrolysate | 246.4 | 98.82 | 11.20 | 2.93 | Whole-cell catalysis. | Air | This study |

**Table S2.** Strains, plasmids and primers used in this study

| **Strain** | **Description** | **Reference** |
| --- | --- | --- |
| *E. coli* DH5α | F-, ф80, lacZ∆M15, ∆(lacZYA-argF), U169, endA1, recA1, hsdR17(rk-mK+), supE44, λ−, thi-1, gyrA96, relA1, phoA | Tiangen, Beijing, China |
| *E. coli* HB101 | supE44 hsdS20 (rB− mB−) recA13ara-14 proA2 lacY1 galK2 rpsL20xyl-5 mtl-1, containing pRK2013 | Boyer and Roulland-dussoix, [13] |
| *G. oxydans* DSM2003 | Wild type, Cefr | DSM |
| *G. oxydans*/pBBR | DSM2003，containing pBBR-P_tufb_;Cef^r,^Gm^r^ | This study |
| *G. oxydans*/pBBR-mGDH | DSM2003，containing pBBR-P_tufb_-mGDH; Cef^r^, Gm^r^ | This study |
| *G. oxydans*/pBBR-35-mGDH | DSM2003，containing pBBR-35-P_tufb_-mGDH; Cef^r^, Gm^r^ | This study |
| *G. oxydans*/pBBR-10-mGDH | DSM2003，containing pBBR-10-P_tufb_-mGDH; Cef^r^, Gm^r^ | This study |
| *G. oxydans*/pBBR-R35-mGDH | DSM2003，containing pBBR-R35-P_tufb_-mGDH; Cef^r^, Gm^r^ | This study |
| *G. oxydans*/pBBR-R10-mGDH | DSM2003，containing pBBR-R10-P_tufb_-mGDH; Cef^r^, Gm^r^ | This study |
| *G. oxydans*/pBBR-3510-mGDH | DSM2003，containing pBBR-3510-P_tufb_-mGDH; Cef^r^, Gm^r^ | This study |
| *G. oxydans*/pBBR-R3510-mGDH | DSM2003，containing pBBR-R3510-P_tufb_-mGDH; Cef^r^, Gm^r^ | This study |
| **Plasmid** | **Description** | **Reference** |
| pBBR1MCS5 | Gm^r^ | Kovach et al., [14] |
| pRK2013 | Helper plasmid, Km^r^ | ATCC |
| pBBR-35 | The derivate of pBBR1MCS5, Gm^r^ | Shi et al., [15] |
| pBBR-10 | The derivate of pBBR1MCS5, Gm^r^ | Shi et al., [15] |
| pBBR-R35 | The derivate of pBBR1MCS5, Gm^r^ | Shi et al., [15] |
| pBBR-R10 | The derivate of pBBR1MCS5, Gm^r^ | Shi et al., [15] |
| pBBR-3510 | The derivate of pBBR1MCS5, Gm^r^ | Shi et al., [15] |
| pBBR-R3510 | The derivate of pBBR1MCS5, Gm^r^ | Shi et al., [15] |
| pBBR-P_tufb_ | pBBR1MCS5 containing promoter tufb from *G. oxydans* 621H | This study |
| pBBR-P_tufb_-mGDH | pBBR1MCS5 derivate expressing mgdh under the control of promoter tufb | This study |
| pBBR-35-P_tufb_-mGDH | pBBR-35 derivate expressing mgdh under the control of promoter tufb | This study |
| pBBR-10-P_tufb_-mGDH | pBBR-10 derivate expressing mgdh under the control of promoter tufb | This study |
| pBBR-R35-P_tufb_-mGDH | pBBR-R35 derivate expressing mgdh under the control of promoter tufb | This study |
| pBBR-R10-P_tufb_-mGDH | pBBR-R10 derivate expressing mgdh under the control of promoter tufb | This study |
| pBBR-3510-P_tufb_-mGDH | pBBR-3510 derivate expressing mgdh under the control of promoter tufb | This study |
| pBBR-R3510-P_tufb_-mGDH | pBBR-R3510 derivate expressing mgdh under the control of promoter tufb | This study |
| **Primer** | **Sequences of Primer (5‘-3’)** | **Restriction** **endonuclease^a^** |
| tufb-f | ACTGAGCTCCGATGGTAAGAAATCCACTGC | *Sac*I |
| tufb-r | ATATCTAGACCAAAACCCCGCTCCACC | *Xba*I |
| gdh-f | AACTCTAGAAGGAACATCATGAGCACAAC | *Xba*I |
| gdh-r | GAGGAATTCGTCAGATCATTTCTGATCG | *EcoR*I |
| q16s-f | GCGGTTGTTACAGTCAGATG |  |
| q16s-r | GCCTCAGCGTCAGTATCG |  |
| qGox0265-f | AGATCAACGCGTCCAACGTC |  |
| qGox0265-r | CACCATCAACCGCAAACAGC |  |

^a^ Restriction endonuclease sites underlined


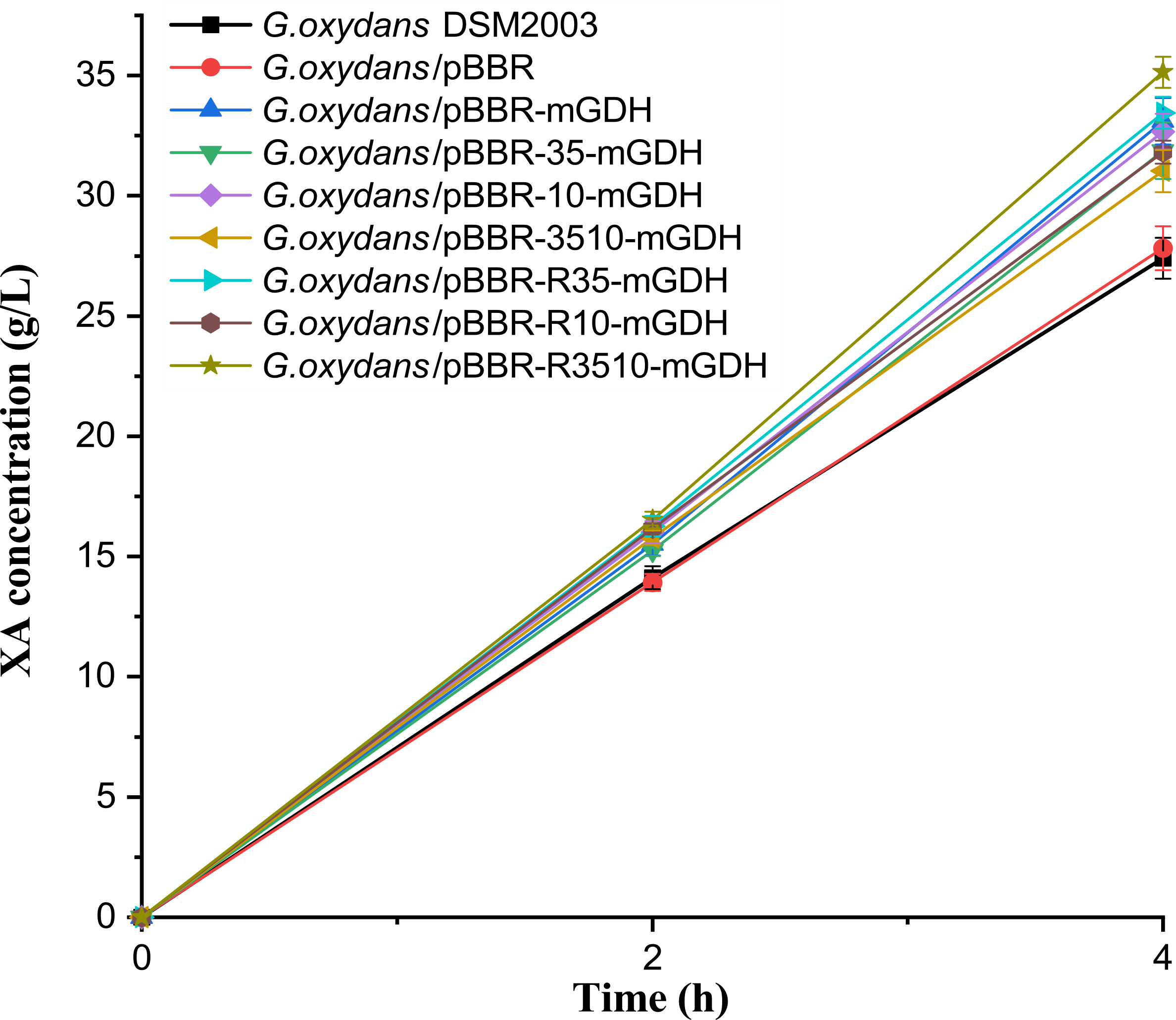


**Figure S1.** Comparison of D-xylonic acid production by different *G. oxydans* strains.

The conversions were carried out in shake flasks at 30°C and 200 rpm. The reaction system contained 40 g/L D-xylose, 50 mM citric acid buffer (pH 5.8), and 1.90 g_dcw_/L resting cells.





**Figure S2.** The activities of mGDHs from *G. oxyd*ans DSM2003 and *G. oxydans*/pBBR-R3510-mGDH toward D-xylose.


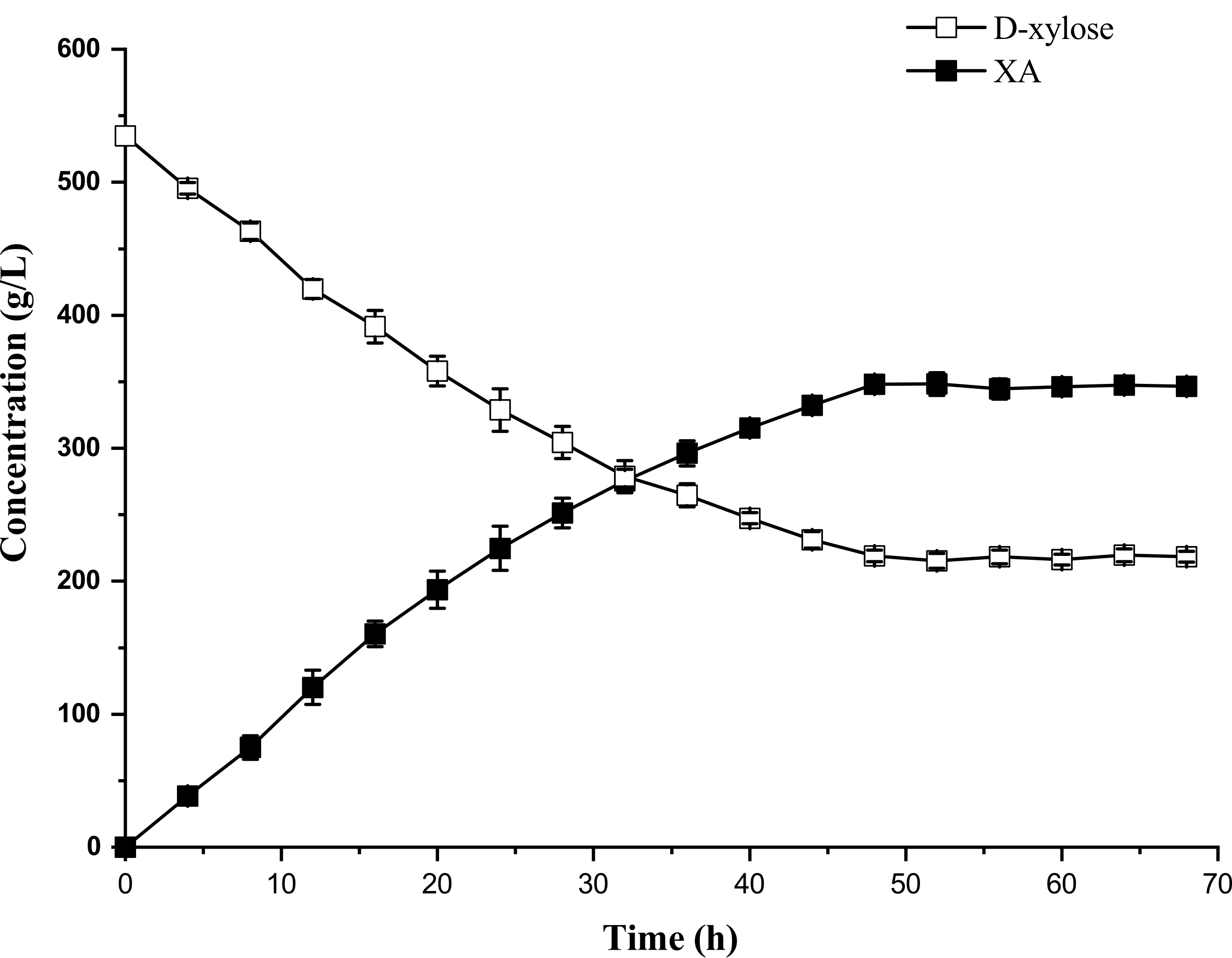


**Figure S3.** Batch D-xylonic acid production from 535 g/L D-xylose by *G. oxydans*/pBBR-R3510-mGDH.

The conversion was carried out in a 7 L fermenter with 4 vvm aeration, 30°C and 600 rpm conditions. The pH was maintained at 5.8 by using a 4 M NaOH solution. The reaction system contained 535 g/L D-xylose and 3.82 g_dcw_/L resting cells.


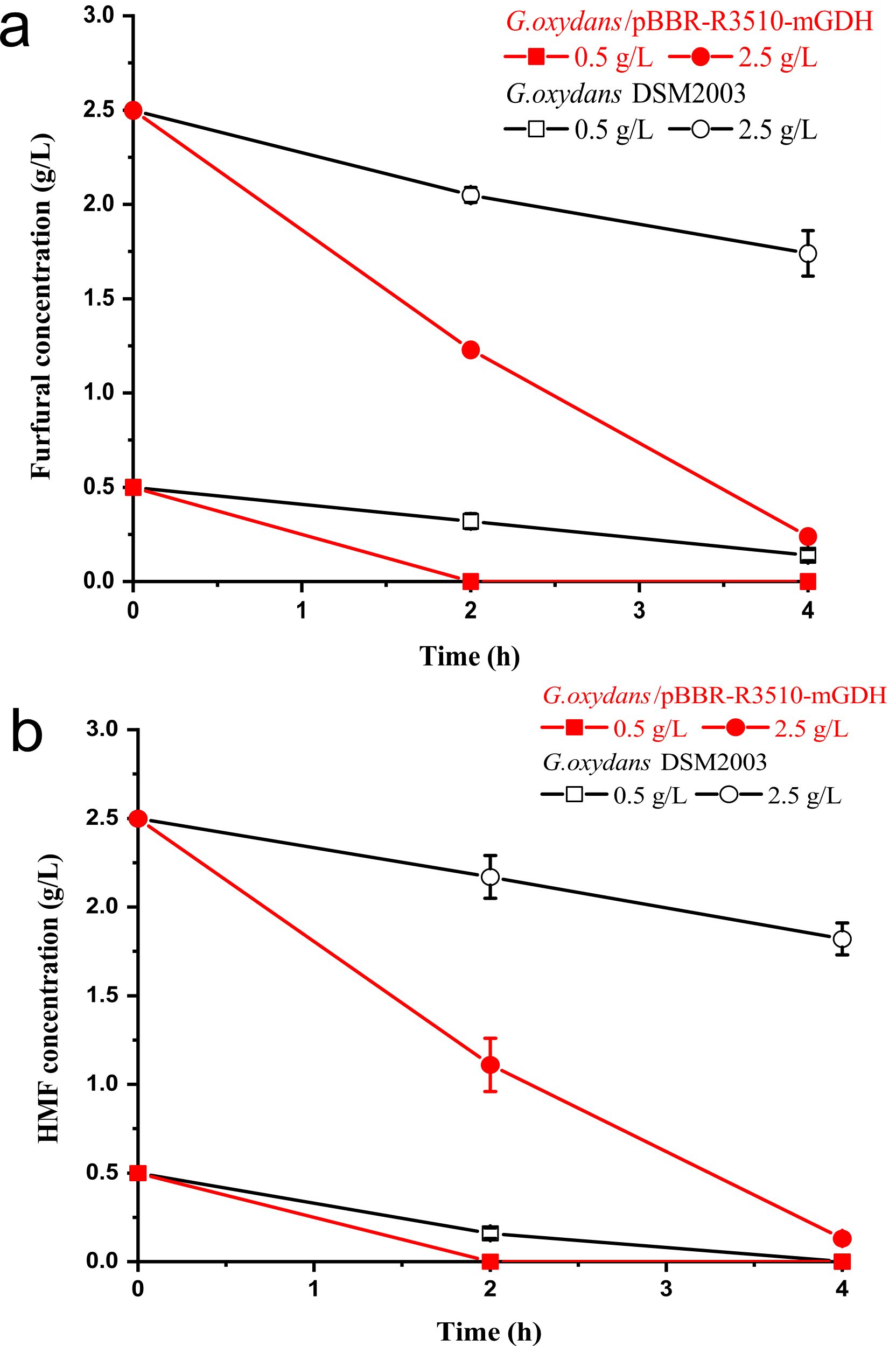


**Figure S4.** Transformation of furfural and HMF by *G. oxydans*/pBBR-R3510-mGDH and *G. oxydans* DSM2003, respectively. (a) Transformation of furfural; (b) transformation of HMF. The conversions were carried out in shake flasks at 30°C and 200 rpm. The reaction system contained 0.5 (2.5) g/L furfural (HMF), 40 g/L D-xylose, 50 mM citric acid buffer (pH 5.8), and 1.90 g_dcw_/L resting cells.





**Figure S5.** The activities of the membrane-bound dehydrogenases of membrane fractions from *G. oxydans* DSM2003 and *G. oxydans*/pBBR-R3510-mGDH.

**Methods**

***Preparation of membrane fractions***

*G. oxydans* cells were cultivated in sorbitol medium at 30°C and harvested at the late exponential phase. The resting cells were washed twice with ice-cold 50 mM sodium phosphate buffer (pH 7.5) containing 1 mM dithiothreitol and 1 mM MgCl_2_. The washed cells were resuspended in the same buffer at 50 g_wet wt._/L and passed twice through high-pressure homogenization at 80 MPa. After a centrifugation at 8000 × g for 30 min to remove the cell debris, the supernatants were centrifuged at 15000 × g at 4°C for 90 min. The precipitate was subsequently resuspended in 10 mM sodium phosphate buffer (pH 6.0) and used as the membrane fraction [16]. The protein concentration was measured by the Bradford method [17].

***Enzyme assay***

The activity of membrane-bound dehydrogenase was measured by the initial reduction rate of 2,6-dichlorophenolindophenol at 600 nm and 30°C. The basal reaction mixture (200 μL) for the enzyme assay contained 50 mM sodium phosphate buffer (pH 6.0), 2 mM 2,6-dichlorophenolindophenol, 2.6 mM phenazine methosulfate, and 0.03 mg of membrane protein. The reactions were initiated by adding 50 mM substrates (furfural, HMF and D-xylose) and were incubated at 30°C for 5 min. One unit of dehydrogenase activity was defined as the amount of the enzyme that catalyzes the reduction of 1 μM 2,6-dichlorophenolindophenol per min at 30°C. The molar extinction coefficient of 2,6-dichlorophenolindophenol at pH 6.0 was 10.8 mM^−1^ [18].

**Supplementary references**

1. Zhang H, Liu G, Zhang J, Bao J. Fermentative production of high titer gluconic and xylonic acids from corn stover feedstock by *Gluconobacter oxydans* and techno-economic analysis. Bioresour Technol. 2016;219:123-31.

2. Zhou X, Lü S, Xu Y, Mo Y, Yu S. Improving the performance of cell biocatalysis and the productivity of xylonic acid using a compressed oxygen supply. Biochem Eng J. 2015;93:196-9.

3. Dai L, Jiang W, Zhou X, Xu Y. Enhancement in xylonate production from hemicellulose pre-hydrolysate by powdered activated carbon treatment. Bioresour Technol. 2020;316:123944.

4. Bondar M, da Fonseca MMR, Cesário MT. Xylonic acid production from xylose by *Paraburkholderia sacchari*. Biochem Eng J. 2021;170:107982.

5. Dvořák P, Kováč J, de Lorenzo V. Biotransformation of d-xylose to d-xylonate coupled to medium-chain-length polyhydroxyalkanoate production in cellobiose-grown *Pseudomonas putida* EM42. Microb Biotechnol. 2020;13:1273-83.

6. Ji H, Lu X, Zong H, Zhuge B. A synthetic hybrid promoter for D-xylonate production at low pH in the tolerant yeast *Candida glycerinogenes*. Bioengineered. 2017;8:700-6.

7. Buchert J, Viikari L. The role of xylonolactone in xylonic acid production by Pseudomonas fragi. Appl Microbiol Biotechnol. 1988;27:333-6.

8. Toivari M, Vehkomäki ML, Nygård Y, Penttilä M, Ruohonen L, Wiebe MG. Low pH D-xylonate production with *Pichia kudriavzevii*. Bioresour Technol. 2013;133:555-62.

9. Sundar MSL, Susmitha A, Rajan D, Hannibal S, Sasikumar K, Wendisch VF, et al. Heterologous expression of genes for bioconversion of xylose to xylonic acid in *Corynebacterium glutamicum* and optimization of the bioprocess. AMB Express. 2020;10:68.

10. Gao C, Hou J, Xu P, Guo L, Chen X, Hu G, et al. Programmable biomolecular switches for rewiring flux in *Escherichia coli*. Nat Commun. 2019;10:3751.

11. Zhang Y, Guo S, Wang Y, Liang X, Xu P, Gao C, et al. Production of d-xylonate from corn cob hydrolysate by a metabolically engineered *Escherichia coli* strain. ACS Sustain Chem Eng. 2019;7:2160-8.

12. Toivari M, Nygård Y, Kumpula EP, Vehkomäki ML, Benčina M, Valkonen M, et al. Metabolic engineering of *Saccharomyces* cerevisiae for bioconversion of D-xylose to D-xylonate. Metab Eng. 2012;14:427-36.

13. Boyer HW, Roulland-Dussoix D. A complementation analysis of the restriction and modification of DNA in *Escherichia coli*. J Mol Biol. 1969;41:459-72.

14. Kovach ME, Elzer PH, Hill DS, Robertson GT, Farris MA, Roop RM, 2nd, et al. Four new derivatives of the broad-host-range cloning vector pBBR1MCS, carrying different antibiotic-resistance cassettes. Gene. 1995;166:175-6.

15. Shi YY, Li KF, Lin JP, Yang SL, Wei DZ. Engineered expression vectors significantly enhanced the production of 2-Keto-D-gluconic acid by *Gluconobacter oxidans*. J Agric Food Chem. 2015;63:5492-8.

16. Meyer M, Schweiger P, Deppenmeier U. Effects of membrane-bound glucose dehydrogenase overproduction on the respiratory chain of *Gluconobacter oxydans*. Appl Microbiol Biotechnol. 2013;97:3457-66.

17. Bradford MM. A rapid and sensitive method for the quantitation of microgram quantities of protein utilizing the principle of protein-dye binding. Anal Biochem. 1976;72:248-54.

18. Zhang H, Shi L, Mao X, Lin J, Wei D. Enhancement of cell growth and glycolic acid production by overexpression of membrane-bound alcohol dehydrogenase in *Gluconobacter oxydans* DSM 2003. J Biotechnol. 2016;237:18-24.
